# Cerebral Venous Thrombosis as an Extrahepatic Manifestation of Acute Anicteric Hepatitis A Infection

**DOI:** 10.1155/2012/120423

**Published:** 2012-07-17

**Authors:** Panagiotis Zis, Elli Kontogeorgi, Dimitrios Karakalos, Despoina Pavlopoulou, Vassilios A. Sevastianos

**Affiliations:** ^1^Department of Neurology, Evangelismos General Hospital, 10676 Athens, Greece; ^2^4th Department of Internal Medicine, Evangelismos General Hospital, 10676 Athens, Greece

## Abstract

Among the many infective causes of cerebral venous thrombosis (CVT), viral hepatitis has been regarded as a rare associated condition. We report the case of a 31-year-old woman presenting with CVT associated with hepatitis A virus (HAV) infection, outlining probable pathogenic mechanisms. We suggest that hepatitis A serological markers should be routinely included in the investigation of cerebral venous thrombosis of unknown etiology, in nonvaccinated patients with risk factors of a recent HAV exposure.

## 1. Introduction

Cerebral venous thrombosis (CVT) is a rare condition, which accounts for <1% of all strokes [1]. As high-quality epidemiologic studies of CVT are lacking, the available data suggest that one can expect five to eight cases per year in a tertiary care centre [2–4]. 

Predisposing causes of CVT are multiple. The risk factors for venous thrombosis in general are linked classically to the Virchow triad of stasis of the blood, changes in the vessel wall, and changes in the composition of the blood. Risk factors are usually divided into acquired risks (e.g., surgery, trauma, pregnancy, puerperium, antiphospholipid syndrome, cancer, and exogenous hormones) and genetic factors (e.g., inherited thrombophilia) [1]. A different way to classify the main recognized causes or predisposing conditions to CVT is to divide them into infectious (either local or systemic) and noninfectious causes [5].

We report for the first time in the bibliography a rare case of a patient with CVT as the sole extrahepatic clinical manifestation of acute anicteric hepatitis A virus (HAV) infection.

## 2. Case

A 31-year-old white woman was admitted to our hospital with a 48-hour history of worsening headache and vomiting. She had no previous history of headaches. Three days prior to her admission she returned from a short trip to her home country. She had no past medical history and was a nonsmoker without any history of drug or alcohol abuse. She had one child and was not currently using any contraceptives. Her one delivery had been without complication and she had never had a termination of pregnancy.

General physical examination was unremarkable. Neurological examination revealed right hemiparesis and total aphasia. Routine blood and urine exams were unremarkable, apart from an elevated c-reactive protein (CRP = 5.6 mg/dL, normal values = 0–0.5 mg/dL).

**Figure 1:**
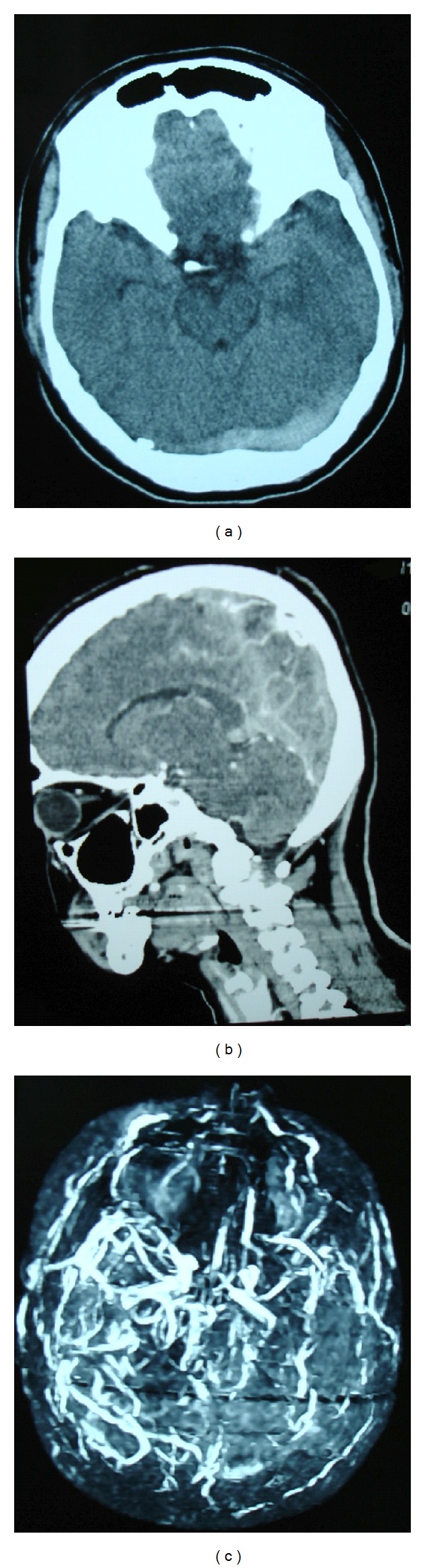
Cranial computerized tomography indicating left transverse sinus thrombosis. (b) Computerized tomography angiogram indicating superior sagittal and straight sinus thrombosis. (c) Magnetic resonance venography indicating the development of collateral circulation following the cerebral venous thrombosis.

Cranial computerized tomography (CT) and computerized tomography angiogram (CTA) indicated left transverse, superior sagittal, and left sigmoid sinus thrombosis associated with left parietal and temporal hemorrhagic venous infarcts (Figures 1(a) and 1(b)). Magnetic resonance venography (MRV) confirmed the above findings (Figure 1(c)). Further blood samples were taken in order to investigate possible secondary aetiologies and at the same time subcutaneous low molecular weight heparin was initiated.

Values of serum immunoglobulins were IgA = 180 mg/dL (normal values 72–400 mg/dL), IgG = 1050 mg/dL (normal values 690–1618 mg/dL), and IgM = 306 mg/dL (normal values 40–235 mg/dL). The following results from blood immunology were negative : antinuclear antibodies (abs), anti-ds-DNA abs, anti-Sm abs, antimitochondrial abs, IgM and IgG anticardiolipins, anticitrullinated protein, rheumatoid factor, p-ANCA, c-ANCA, and anti-TPO abs. Serum complement values were within the normal range and cryoglobulins were not detected in the serum.

The following results from blood serology were negative: Rapid Plasma Reagin (RPR), HBsAg, anti-HBc, anti-HBs, anti-HBe, HBeAg, antiHBc IgM, anti-HCV, anti-HDV, and HIV. Thrombophilic states were sought but antithrombin III, factor V Leiden, PCGlob-FVNR, and protein C and S levels were within normal limits. The molecular genetic screening for thrombophilia revealed that the patient was heterozygous for the 20210G > A and the MTHFR C677T mutation.

**Figure 2:**
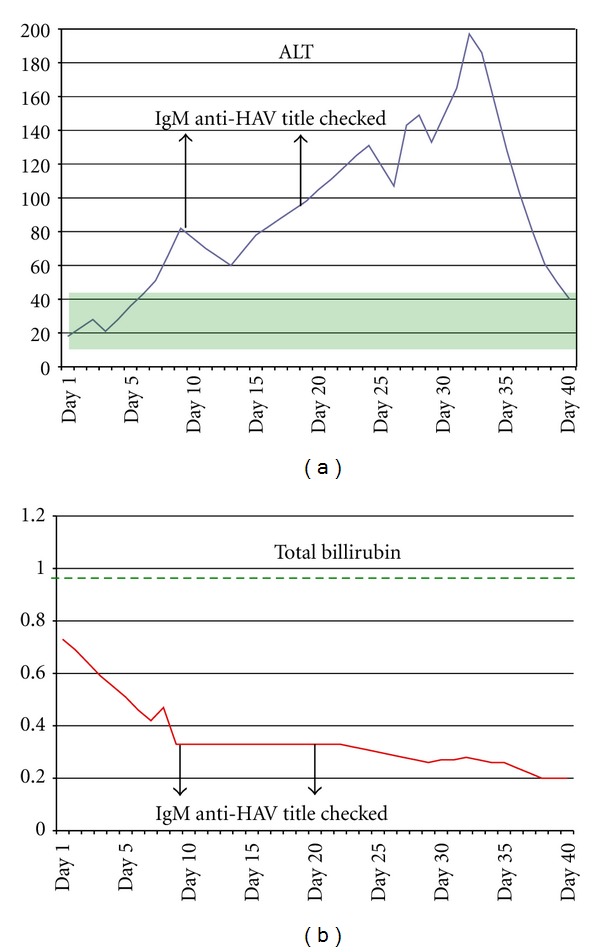
(a) Alanine transaminase level over days of hospitalization, in IU/L (normal values 5–40 IU/L). (b) Total billirubin level over days of hospitalization, in mg/dL (normal values 0-1 mg/dL).

Anticoagulant treatment with warfarin was initiated (target INR 2-3) and low molecular weight heparin was stopped. On Day 10 of her hospital admission, she complained of right upper quadrant abdominal pain and vomiting. Moreover, the liver enzymes gradually increased (Figure 2(a)), with no subsequent increase of the total billirubin (Figure 2(b)), and it was difficult to maintain the target INR, as even with very small warfarin doses (0,25–0,50 mgs) the INR was persistently elevated (4.0–4.5). Further investigation of her abnormal liver function revealed serum IgM antibodies to HAV in high titles (≥1,21 RLU-reactive result, by Architect system, repeated twice), related to recent viral exposure.

Conservative treatment was continued and the liver enzyme levels returned to normal. Patient serological reassessment five months later showed, as expected, the emergence of IgG antibody to HAV and decreased serum levels of IgM antibody. Anticoagulation with warfarin was maintained for six months and then suspended. General examination is unremarkable and neurological examination now reveals only right arm weakness. The patient signed informed consent to allow her personal data publication.

## 3. Discussion

The existing studies suggest that the approximate incidence of CVT is between 0.22 and 1.23/100.000 per year [6, 7]. The peak incidence in adults is in their third decade with a male/female ratio of 1 : 3 [8, 9].

The pathogenesis of CVT remains incompletely understood because of the high variability in the anatomy of the venous system and the paucity of experiments in animal models of CVT. However, two possible mechanisms may contribute to the clinical features of CVT; thrombosis of cerebral veins or dural sinus leading to cerebral parenchymal lesions or dysfunction and occlusion of dural sinus resulting in decreased cerebrospinal (CSF) absorption and elevated intracranial pressure [10].

CVT has a highly variable clinical presentation [2]. Symptoms and signs of CVT can be grouped in three major syndromes; (a) isolated intracranial hypertension syndrome (headache with or without vomiting, papilloedema, and visual problems) [11], (b) focal syndrome (focal deficits, seizures, or both), and (c) encephalopathy (multifocal signs, mental state changes, stupor, or coma) [2]. 90% of patients with sinus thrombosis suffer from headache, which tends to worsen over a period of several days, or develops suddenly [12].

In 1999 a case of a 43-year-old male patient with CVT associated with hepatitis B was presented [13]. Moreover, in 2006, a case of a 56-year-old male patient with presenting CVT associated with hepatitis B and C coinfection was reported [5]. To our knowledge, we have reported for the first time a case of patient who suffered from CVT a few days after the onset of HAV infection. The history of the recent trip to a country with a high prevalence of HAV, the characteristic clinical picture of the patient, the abnormal liver enzymes, the absence of hypergammaglobulinaemia, and the presence of serum IgM antibodies to HAV are strongly supportive of a recent infection and an initial acute phase of the disease. Moreover, the fact that the rheumatoid factor was not present minimizes the possibility of a false positive result regarding the IgM antibodies to HAV [14, 15].

HAV is spread via the faecal-oral route, and is more prevalent in lower socioeconomic areas where poor hygiene practices and a lack of adequate sanitation spread the infection. The incubation period averages 30 days (range 15 to 49 days), after which the illness begins with the abrupt onset of prodromal symptoms including fatigue, malaise, nausea, vomiting, anorexia, fever, and right upper quadrant pain [16]. HAV infection usually results in an acute, self-limiting illness and only rarely leads to fulminant hepatic failure [17].

Infections may trigger the thrombosis directly by causing septic thrombosis or indirectly by precipitating thrombosis in people who suffer from a prothrombotic illness [18]. In a study designed to assess the association of prothrombotic factors and underlying conditions (including infections, vascular trauma, immobilization, malignancies, autoimmune diseases, renal diseases, metabolic disorders, obesity, cardiac malformations, and use of prothrombotic drugs) with CVT in children it was concluded that CVT in children is a multifactorial disease that, in the majority of cases, results from a combination of prothrombotic risk factors and/or an underlying clinical condition [19]. In a series of 42 patients with CVT, more than half of them had recent infections, a fact that emphasizes that people with prothrombotic conditions may develop thrombosis after having any systemic infection [20].

There is growing evidence that hepatitis B and C viruses alone or in combination with a series of other factors may shift the delicate procoagulant/thrombolysis balance towards thrombosis [21]. Extrahepatic manifestations of hepatitis A are uncommon and are usually immunologically mediated. Relapsing hepatitis A seems to be associated with a higher incidence of immune-related clinical and laboratory phenomena. Cutaneous vasculitis, with immunoglobulin M deposits and vasculitis cryoglobulinaemia and cholecystitis have been reported, along with neurological syndromes such Guillain-Barré, myelopathy, mononeuritis, meningoencephalitis or exacerbations of pre-existing multiple sclerosis [22]. In our case, CTV as the sole manifestation of anicteric acute hepatitis A infection should not be discarded.

Therefore, we suggest that hepatitis A serological markers should be routinely included in the investigation of cerebral venous thrombosis of unknown etiology, in non-vaccinated patients with risk factors of a recent HAV exposure.
